# An investigation into the relationship between nutritional status, dietary intake, symptoms and health-related quality of life in children and young people with juvenile idiopathic arthritis: a systematic review and meta-analysis

**DOI:** 10.1186/s12887-022-03810-4

**Published:** 2023-01-02

**Authors:** Najmeh Zare, Maedeh Mansoubi, Shelly Coe, Ali Aminalsharieh Najafi, Kathryn Bailey, Kathryn Harrison, Joanna Sheehan, Helen Dawes, Karen Barker

**Affiliations:** 1 Paediatric Nursing, Centre for Movement, Occupational and Rehabilitation Sciences, Oxford Brookes University, Oxford, United Kingdom; 2 College of Medicine and Health, University of Exeter, Exeter, United Kingdom; 3 Centre for Movement, Occupational and Rehabilitation Sciences, Oxford Institute of Nursing, Midwifery and Allied Health Research, Oxford Brookes University, Oxford, United Kingdom; 4 Oxford Clinical Allied Technology and Trial Services Unit (OxCATTS), Oxford, United Kingdom; 5 Oxford Brookes Centre for Nutrition and Health, Oxford Brookes University, Oxford, United Kingdom; 6 Health and Social Care, Centre for Movement, Occupational and Rehabilitation Sciences, Oxford Brookes University, Oxford, United Kingdom; 7 Consultant Paediatric Rheumatology, Oxford Health NHS Foundation Trust, Oxford, United Kingdom; 8 Consultant Paediatric Rheumatology, Birmingham Women’s and Children’s NHS Foundation Trusts, Birmingham, United Kingdom; 9 BSc Physiotherapy Clinical Specialist Physiotherapist in Paediatric Rheumatology, Oxford University Hospital NHS Foundation Trust, Oxford, United Kingdom; 10 Professor Of Clinical Rehabilitation, College of Medicine and Health, University of Exeter, Exeter, United Kingdom; 11 NIHR Oxford Health Biomedical Research Centre, Oxford, United Kingdom; 12 Professor of Physiotherapy NDORMS, University of Oxford, Oxford, United Kingdom

**Keywords:** Juvenile idiopathic arthritis, Vitamin D, Diet, Symptoms, Health-related quality of life, Weight, Height

## Abstract

**Background:**

The association between diet, symptoms and health related quality of life in children and young people with Juvenile idiopathic arthritis (JIA) is not clearly understood. The objectives of this systematic review and meta-analysis were to explore the evidence for a relationship between nutritional status, dietary intake, arthritis symptoms, disease activity and health-related quality of life in children and young people with JIA considering both observational and interventional studies separately.

**Method:**

The databases PubMed, CINAHL, PsycINFO, Web of Science and Cochrane were searched in October 2019, updated in September 2020 and October 2021. Searches were restricted to English language, human and age (2–18 years old). Studies were included if they measured the effect of dietary supplements, vitamins or minerals, or diet in general, on quality of life and/ or arthritis symptom management. Two researchers independently screened titles and abstracts. Full texts were sourced for relevant articles. PRISMA guidelines were used for extracting data. For variables (vitamin D and disease activity), a random-effects meta-analysis model was performed. Two authors using a standardized data extraction form, extracted data independently.

**Results:**

11,793 papers were identified through database searching, 26 studies met our inclusion criteria with 1621 participants. Overall studies quality were fair to good. Results from controlled trial and case control studies with total 146 JIA patients, found that Ɯ-3 PUFA improved the mean active joint count (*p* < 0.001), Juvenile Arthritis Disease Activity Score (JADAS-27) (*p* < 0.001) and immune system (≤ 0.05). Furthermore, n-3 and n-6 PUFAs have a negative correlation with CRP (C-reactive protein) and ESR (erythrocyte sedimentation rate) (*p* < 0.05). Improvement in JIA symptoms were observed in one case, one pilot and one exploratory study with overall 9 JIA patients after receiving Exclusive Enteral Nutrition (EEN) which contains protein and what is required for a complete nutrition, A clinical trial study found Kre-Celazine nutrition (composed of a proprietary alkali buffered, creatine monohydrate and fatty acids mixture) in 16 JIA patients improved symptoms of JIA. No association was found between vitamin D and disease activity from three studies. Height and weight values in relation to healthy controls varied across studies (*p* = 0.029).

**Conclusions:**

We were only able to include small studies, of lower design hierarchy, mainly pilot studies. We found some evidence of lower height and weight across studies in JIA, but were unable to confirm an association between diet, symptoms and health-related quality of life in children and young people with JIA. Well-designed, carefully measured and controlled interventional studies of dietary patterns in combination with important contributing factors such as medication and lifestyle behaviours, including physical activity, are required to determine the impact of diet in improving symptoms and growth patterns in children and young people with JIA, with an aim to improve the quality of their life.

**Trial registration:**

PROSPERO [CRD42019145587].

**Supplementary Information:**

The online version contains supplementary material available at 10.1186/s12887-022-03810-4.

## Introduction

Juvenile idiopathic arthritis (JIA) is the most prevalent autoimmune rheumatic disease in the paediatric population, with a prevalence of approximately one in 1000 [1, 2] and a significant cause of short and long-term disabilities [3]. Young people with JIA have a poorer Health-related quality of life (HRQOL) as compared with healthy peers related to the chronic relapsing nature of the condition, unpredictable disease course, symptom management and long-term treatments [4–7]. The cause of JIA is still unknown, yet it is likely to involve both genetic susceptibility and environmental factors [8]. Lifestyle factors, including diet, are important determinants of health and wellbeing in young people living with JIA, with the implementation of nutrition interventions including vitamin D suggested to potentially regulate disease severity and associated symptoms through an effect on the immune system [9]. Recently it has also been suggested that vitamin D is an environmental factor that, affects the prevalence of autoimmune diseases by modulating the immune system [10]. Whilst, the potential mechanism has been highlighted, the influence of vitamin D dosage in both the development of JIA and disease activity is still unclear [11]. The impact of diet and nutrition is difficult to interpret in the paediatric population, particularly those with rheumatic disease, due to family influence, dietary regulation and data collection in children [12, 13]. Suboptimal nutrition is suggested to adversely affect the long-term outcome of this group of children and is a source of considerable concern to parents and patients alike [11]. Nutritional deficits affect the general well-being of the child, may adversely affect disease control and contribute to growth disturbance. Considering the potential benefits of diet on short and long-term health and wellbeing, a better understanding of optimal dietary approaches is of value and could inform the development of management strategies. Despite the importance of diet and its potential impact on the HRQOL in children and young adults with JIA, the dietary factors associated with better outcomes are not clearly understood, with the literature varying in quality and including heterogeneous outcomes. The overall aim of this systematic review and meta-analysis is to assess the quality and extent of the evidence of the relationship between nutritional status, dietary intake, arthritis symptoms, disease activity and health-related quality of life in children and young people with JIA considering both observational and interventional studies separately.

## Methods

This study was performed following Preferred Reporting Items for Systematic Review and Meta-Analysis guidelines [14]. The protocol for this review was registered with PROSPERO [CRD42019145587].

### Data sources and searches

**Fig. 1:**
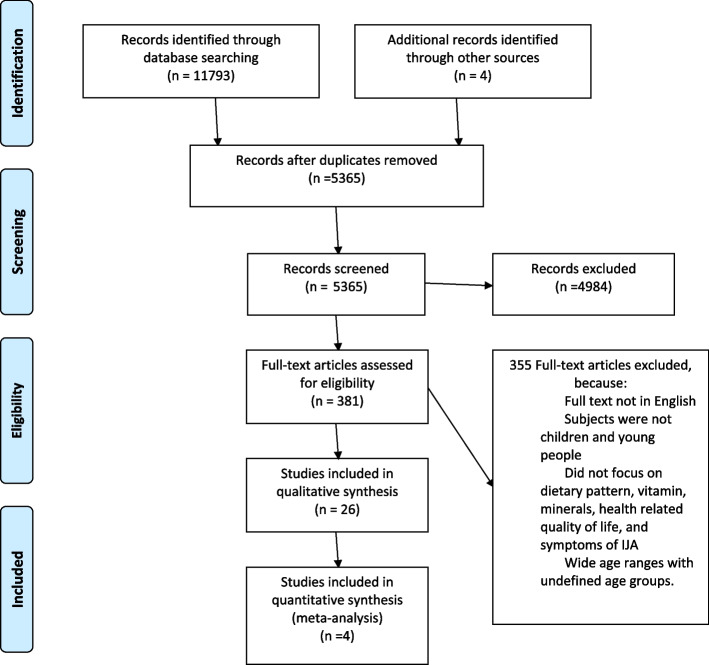
Study selection flow diagram

The following databases were used from inception to October 2019 to conduct searches: PubMed, CINAHL, Cochrane, PsycINFO and Web of Science. Search was updated in September 2020 and October 2021. The full search strategy is included in Fig. 1. Six researchers were involved in this systematic review. Two researchers independently screened titles and abstracts. Full texts were sourced for relevant articles. Inclusion criteria were assessed independently.

### Eligibility criteria

Observational research, including baseline intervention or control arm data, longitudinal, control trial, randomised controlled trial and case studies were included in this systematic review and meta-analysis. Studies were included that measured the effect of dietary patterns, vitamin/mineral levels or supplements on disease activity, symptoms such as fatigue, pain, inflammation and quality of life in JIA. Searches were restricted to English language publications, human and age (2–18 years old) but no date restrictions were imposed. These criteria are derived from the literature review and consensus opinion of experts.

### Data extraction

Two authors using a standardized data extraction form extracted data independently. Extracted data included study characteristics, baseline demographics of participants, description of the intervention, subtypes of arthritis, measurement, interventions, primary and secondary outcomes.

Outcomes. The primary outcome of this systematic review and meta-analysis was to assess the evidence of the extent of the relationship of dietary intake (patterns, vitamins/mineral levels or supplements) on the quality of life in children and adolescents with JIA. The additional outcome was to assess the evidence of the extent of the effect of dietary supplements or diet on disease activity and symptoms of arthritis, including pain, fatigue, joint stiffness, inflammation, weight and height.

Assessment of risk of bias and quality of evidence. Two researchers independently assessed the quality of all included trials by using the Cochrane Collaboration risk of bias tool [15]. If two of the domains were rated as high, the study was considered to be at high risk of bias. The NIH quality assessment tool was applied to assess the quality of observational cohorts, case–control, case series and cross-sectional studies [16]. For the case report, JBI critical appraisal checklist was used [17]. Any discrepancy was resolved over the discussion by another reviewer.

### Data synthesis and analysis

**Fig. 2:**
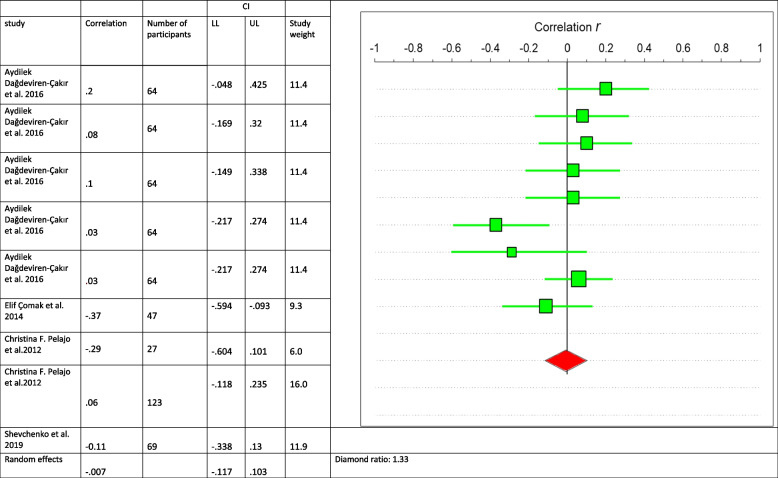
Association Vitamin D and disease activity

A descriptive analysis of each study is provided in Fig. 2. Where four studies reported on a variable, forest plots were used to show the point estimate (95% CIs) for each study. For variables (vitamin D and disease activity), correlation, number of participants, 95%CIs and data weight were calculated using a random-effects meta-analysis model. Standard errors were calculated by converting 95% CIs using the following formula: SD = N × (upper bound of the CI − lower bound of the CI)/3.92 [18].

## Results

### Identification and selection of relevant studies

An overview of the study identification process is provided in Fig. 1. The initial search yielded 11,793 records; after the removal of duplicates and based on title and abstract, 380 papers were retrieved for more detailed evaluation. The full texts of the remaining 355 articles were reviewed against the study selection criteria, and 26 studies that met our inclusion criteria were included.

### Study characteristics

A summary of the characteristics of the selected studies is listed in Tables 1 and 2. Of the 26 selected articles, there were five from the United States, three from Brazil and Sweden, two from Ukraine, Turkey and Finland, and one from Egypt, Bulgaria, Poland, Italy, Norway, United Kingdom, Australia, Morocco and China. Seven were cross-sectional (601participants), seven were case controls (526 participants), three were randomised control trials (153 participants), one case study, one pilot study with one participant, one cross-sectional cohort with 80 participants, one clinical trial with 16 participants, two control trials with 69 participants, two cross-sectional studies with control group with 167 participants and one exploratory study with seven participants. The most recent classification system International League of Associations for Rheumatology (ILAR) was used in 15(57.6%) studies to identify arthritis, while three studies used the American college of rheumatology (ACR), and the remaining studies did not mention their classification. In the majority of patient populations, there were greater numbers of females. Oligoarticular JIA was the most common disease category reported across all the studies.

**Table 1:** characteristics of the selected studies (trials, exploratory, case and pilot studies)

Author/ Year	Number of the participants	Study method	Diet/supplements intervention	primary outcome (findings)
Tang et al. 2019 [19]	Total:36 JIA:18 Control:18	Randomised control trial	Cholecalciferol Vitamin D supplementation	Higher levels of serum 25OHD: *P* < 0.05 JADAS-27 was not significant: *P* > 0.05 No correlation between vitamin D, disease activity and BMD
Stark et al. 2006 [20]	Total:49 BI group:25 ESC group:24	Randomised clinical trial	Group 1: JRA received the 6-session BI increased to increase taking dietary calcium Group 2: JRA received 3-session ESC to increase Ca	Ca was significantly greater in the BI group (P < .001) 25-OH-D concentration did not differ between or within intervention group (p > .05)
Dilandro et al. 2015 [12]	Total:22 JIA:12 Control:10	Controlled trial	Biscuit supplementation contains iron	Decreased inflammatory indexes (particularly ESR) *p* = 0.002 No differences in changes were observed for serum iron and ferritin. (*p* = 0.621, 0.166 respectively)
Golini and Jones. 2014 [21]	Total:16	Clinical trial	Kre-Celazine nutritional Supplement composed of a proprietary alkali buffered creatine monohydrate and acetylated fatty acids mixture	Normal range of motion Decreased ESR and CRP Decreased pain scores
Gheita et al. 2012 [22]	Total:47 JIA:27 Control:20	Controlled trial	Dietary supplements of omega-3 FAs	A remarkable improvement in: mean active joint count (*p* < 0.001) JADAS-27 (*p* < 0.001) CHAQ (*p* < 0.001) A significant reduction in the ESR
Yarema et al. 2018 [23]	Total:68 JIA:53 Control:15	Randomised controlled trial	Dietary supplements of omega-3 PUFA	Immune status significantly improved:(≤ 0.05)
Berntson et al.2014 [24]	1	case study	Full nutrition by EEN	Improvement in: global assessment of pain (VAS 0–100 mm) CHAQ JADAS 27
Berntson et al.2016 [24]	1	Pilot study	Full nutrition by EEN	A remarkable Improvement in: number of inflamed joints, morning stiffness, global assessment of pain (VAS 0–100 mm) CHAQ
Berntson et al.2016 [24]	7	Exploratory study	Full nutrition by EEN	An immediate anti-inflammatory effect, as shown by reduction in inflammatory Proteins MCP-2, MCP-3 and MCP-4 (monocyte chemoattractant protein) resulting in clinical improvement in patients with JIA and decreased in levels of JADAS27, CHAQ and pain VAS

**Table 2:** characteristics of the selected studies (case–control, cross-sectional, cross-sectional with control and cross-sectional cohort)

**Author/ year**	**Number of participants**	**Study method**	**primary outcome(findings)**
Shevchenko N., Khadzhynova Y. 2019 [25]	Total:69	Case–control	No correlation between vitamin D status and activity of disease (r-0.11; *P* > 0. 05) No correlation between vitamin D status and number of active joints( r-0.05; *P* > 0.05) No correlation between vitamin D status and number of injured joints (r-0. 14; *P* > 0.05)
Haugen et al. 1992 [26]	Total:32 JCA:15 Control:17	Case–control	Significantly lower weight in PA group than the healthy controls (*p* = 0.01) and pauci-A (*p* = 0.02) Iron and zinc in the PA group compared to Pauci-A (*p* = 0.02) and healthy controls (both *p* < 0.01) Significantly higher disease activity and ESR in the PA group with the pauci-A (*p* = 0.02 and *p* < 0.01 respectively) Positive correlation seen between ESR and the disease activity t (r = 0.54, p < 0.01)
Lofthose et al. 2002 [27]	Total:44 JIA:22 Control:22	Case–control	Significantly lower body fat percentage seen in Pauci-A than in controls (*P* = 0.027)
Honkanen et al. 1990 [28]	Total:137 Control:12 JCA:125	Case–control	chol correlated with the markers of disease activity by ESR and Hb (r = -.193,*p* = 0.002 and r = 0.208,*p* = 0.02) Possessive correlation between serum zinc and chol(r = 0.227,*p* = 0.002) Significant correlation between Zinc and Vit A in serum( *p* < 0.001)
Harper et al. 2000 [29]	Total:78 JRA:44 Control:34	Case–control	JRA Children with TMD reported significantly greater jaw pain, impaired ability to chew, and impaired quality of life before chewing(*P* < 0.05, *P* < 0.001)and after(*P* < 0.05) than did either JRA or control children
Gorczyca et al. 2017 [30]	Total:108 JIA:66 Control:42	Case–control	Negative correlation between n-3 and n-6 PUFAs with CRP and ESR (p < 0.05) Positive correlation between n-3 and n-6 PUFAs and platelet count (*p* < 0.05)
Bacon et al. 1990 [31]	Total:43 JIA:34 Control: 9	Case–control	A significant abnormality was seen in nutritional status in systemic and PA JRA •Zin( p ≤ 05) •Vit A, C and copper: (p ≤ .O1) No significant correlation between diet, nutritional status and growth in any of the three types of JRA
Bouaddi et al. 2014 [10]	Total:40	Cross-sectional	Serum 25(OH) D were associated with DAS28 (*p* = 0.04, β: − 3.87, CI: (− 7.67,-0.07) serum 25(OH)D levels were associated with the following disease activity components: ESR (*p* = 0.05, β: − 0.14, CI: (− 0.28,0.004)), Tender joints (*p* = 0.02, β: − 0.79, CI (− 1.47,-0.10)) Patient global health (*p* = 0.04, β: − 0.17, CI: (− 0.35,-0.004)
Grönlund et al. 2014 [32]	Total:80 JIA:40 Control:40	Cross-sectional cohort	Positive correlation between CHAQ and number of active joints with the proportion of body fat (R 0.48, p 0.002 and R 0.34, p 0.034, respectively)
Gonçalves et al. 2007 [33]	Total:103 JIA:51 Control: 52	Cross-sectional with control group	Average serum Hcy concentration was 9.3 ± 3.16 μmol/L in JIA patients and 8.9 ± 2.42 μmol/L in healthy controls (*p* = 0.615) Vitamin B12 concentration was normal in patients and controls (p < 0.001)
Henderson and Lovell. 1989 [34]	Total:28	Cross-sectional	36% of the patients have PEM 36% were considered not at nutritional risk 28% had some nutritional abnormalities
Amancio et al. 2003 [35]	Total:64 JRA:41 Control:23	cross-sectional with control group	Higher copper levels in male JRA than male in the control group: (*p* = 0.004) Significant relationships between disease activity and the number of inflamed joints with copper levels (*p* = 0.012 and *p* = 0.001, respectively)
Mortensen et al. 1990 [36]	Total:38	cross-sectional	Mean energy intakes were significantly below the RDI in the systemic (*P* = 0.01) and PA(*P* = 0.001) groups Mean intakes of calcium and zinc were below the RDI of 100% in the PA group(*P* = 0.001) The mean intakes for iron, thiamine, niacin equivalents, riboflavin, Vitamins C and A were all above the RDI in each group
Dağdeviren-Çakır et al. 2016 [37]	Total:217 JIA in active disease: 64 JIA in remission: 53 Healthy controls: 100	cross-sectional case–control	Significantly higher levels of Serum 25(OH) vitamin D in healthy subjects compared to the patients (*p* < 0.01) No statistically significant correlation between vitamin D levels and the number of joints with active arthritis (r = 0.1, *p* = 0.4) and physician and family VAS assessments (r = 0.03/*p* = 0.77, r = 0.03/*p* = 0.78 respectively)
Çomak et al. 2014 [38]	Total:47	Retrospective study	A significant negative correlation between 25(OH) D levels and physician VAS, parent VAS and joint count (*p* = 0.001, *p* = 0.001, *p* = 0.02, respectively) A significant negative correlation between 25(OH)D levels and disease activity(*p* = 0.01, r = -0.37)
Caetano et al. 2012 [39]	Total:77 JIA:42 Control:35	Cross-section with controlled group	A significantly greater percentage of total body fat (*p* = 0.001) truncal fat (*p* = 0.011) in JIA girls compared with controls
Pelajo et al. 2012 [9]	Total: 154	Cross-section	No association between 25(OH)D levels and JADAS-27 (*p* = 0.97) Significant associations between JADAS-27 and JIA subtype (*p* = 0.003), and ethnicity (*p* = 0.006)

### Risk of bias

From the randomised trials, one study [20] had an overall low risk of bias, and two had some concern [19, 23], while the remaining trials [12, 21, 22] had an overall high concern. For the cross-sectional studies, overall, four studies had good quality [9, 33, 36, 38] while others were fair [10, 32, 34, 35, 37, 39]. In assessing the risk of bias for case–control studies, two out of six studies had fair quality [28, 30], four were in a good category [26, 27, 29, 31] and one in poor [25]. The pilot and exploratory studies were of good quality [40, 41], and the case study was fair [24] (Table 3).

**Table 3:** Risk of Bias

Qualitative studies					
**Author**	**Type of study**	**tools**	**Quality of study**		
			**Poor**	**fair**	**good**
M-M Grönlund et al	Cross sectional cohort	NIH quality assessment tool		**√**	
Marcela Gonçalves et al	cross-sectional case control study	NIH quality assessment tool			**√**
Carol J. Henderson and Daniel J. Lovell	cross-sectional	NIH quality assessment tool		**√**	
Silverio Amancio et al	cross sectional with control group	NIH quality assessment tool		**√**	
Aydilek Dağdeviren-Çakır et al	cross-sectional case- control	NIH quality assessment tool		**√**	
Elif Çomak et al	cross-sectional Retrospective study	NIH quality assessment tool			**√**
Michelle Cavalcante Caetano et al	Cross sectional, controlled study	NIH quality assessment tool		**√**	
Christina F. Pelajo et al	cross sectional	NIH quality assessment tool			**√**
A. L. MORTENSEN et al	cross-sectional	NIH quality assessment tool			**√**
Ilham Bouaddi	cross-sectional	NIH quality assessment tool		**√**	
**Qualitative studies**					
M. A. Haugen et al	case–control	NIH quality assessment tool			**√**
R.P. Harper et al	case–control	NIH quality assessment tool			**√**
Honkanen er al	case–control	NIH quality assessment tool		**√**	
C. M. Lofthose et al	case–control	NIH quality assessment tool			**√**
Daiva Gorczyca et al	case–control	NIH quality assessment tool		**√**	
Bacon et al	case–control	NIH quality assessment tool			**√**
Shevchenko N., Khadzhynova Y	case–control	NIH quality assessment tool	**√**		
**Trial studies**			**Low concern**	**Some concern**	**High concern**
Tamer Gheita et	Control-trial	Cochrane Collaboration risk of bias tool			**√**
Jeff Golini and Wendy Lou Jones	Clinical trial	Cochrane Collaboration risk of bias tool			**√**
Giancarla Dilandro et. Al	Control trial	Cochrane Collaboration risk of bias tool			**√**
LORI J. STARK et al	Randomized clinical trial	Cochrane Collaboration risk of bias tool	**√**		
Nataliia Yaremaet al	Randomised control trial	Cochrane Collaboration risk of bias tool		**√**	
Tao Tang et al	Randomised control trial	Cochrane Collaboration risk of bias tool		**√**	
**Qualitative studies**					
			**Poor**	**Fair**	**Good**
Berntson, Lillemor et al. 2016 [41]	pilot study	JBI critical appraisal checklist			**√**
Berntson, Lillemor	case study	NIH quality assessment tool		**√**	
Berntson, Lillemor et al. 2016 [41]	Exploratory study	JBI Checklist for Quasi-Experimental Appraisal Tool			**√**

## Results from observational studies

### Nutritional status, vitamin and mineral levels and energy intake in JIA

According to Mortensen [36], mean energy intakes were significantly below the recommended dietary intake (RDI) in the systemic group (*p* = 0.01), and in the polyarticular group mean energy intake, calcium, and zinc were below the RDI (*p* = 0.001). Vitamin C, A and zinc levels for both systemic and polyarticular groups were significantly lower than the control group (*p* ≤ 0.01, *p* ≤ 0.05, *p* ≤ 0.05 respectively) [31], while copper was higher (*p* ≤ 0.05) [36]. There were no significant differences for copper and zinc between juvenile chronic arthritis (JCA) and the control group (*p* = 0.644, *p* = 0.938, respectively), yet in both groups, the mean copper and zinc intakes were below the RDI [36]. Regarding protein–energy malnutrition (PEM), 28% of JIA patients had nutritional abnormalities, which is more prevalent among polyarticular types [34].

Gonçalves and colleagues [33] evaluated plasma homocysteine (Hcy), vitamin B12 and folate in 51 JIA patients and 52 healthy controls. Results were not significant; however, higher average plasma Hcy and folate levels were observed in participants with JIA compared to controls (*p* = 0.615, *p* < 0.001, respectively), whilst there was no difference in vitamin B12 concentration in patients and controls (*p* = 0.341).

Honkanen and others [28], in a case–control study with 137 participants, assessed vitamin A and E levels in juvenile chronic arthritis (JCA). Only vitamin E levels were significantly lower in JCA compared to the control group (*p* < 0.001).

Juvenile idiopathic arthritis and body mass indexes.

Bacon et al. [31] showed a positive correlation between height for age and weight for height (*p* =  + 0.6, P ≤ 0.02) in 14 children with polyarticular arthritis. A negative correlation was found between weight and height in eight patients with systemic arthritis (r =—0.8, *P* < 0.015), yet no correlation was found between these variables in the pauciarticular group with 12 patients.

Haugen and colleagues [26] reported that eight children with polyarticular arthritis had a significantly lower weight compared to both 17 healthy controls (*p* = 0.01) and seven children with pauciarticular arthritis (*p* = 0.02). Similarly, Lofthouse et al. [27] found that in 22 patients with JIA, height and weight were lower compared to healthy control; however, the observed differences were not significant (*p* = 0.096, 0.075, respectively). 15 polyarticular patients had significantly lower weight and height compared with 22 healthy participants (*p* = 0.047 and 0.045 respectively), while the results were not significant in seven pauciarticular JIA (*p* = 0.389, 0.605 respectively).

Mortensen et al. [36] reported that mean height and weight Z scores were significantly below the Z scores in the general population, in the systemic and polyarticular (*p* = 0.009, *p* = 0.02, *P* = 0.001 respectively) groups (*n* = 25). There was no significant difference in the mean weight for height index between the systemic, polyarticular and pauciarticular groups.

Pelajo et al. [9] found in their cross-sectional study that only 28 out of 154 patients with JIA were obese, while Caetano [39] mentioned all 42 girls with JIA had higher median Z-BMI scores (*p* = 0.034) compared with 35 healthy controls. According to Grönlund [33], 40 JIA patients were overweight when compared to 40 matched controls (*p* = 0.029), however, there was no significant difference for height between groups (*p* = 0.11). There were no significant differences in weight and height between 66 JIA patients and 42 healthy controls (*p* = 0.26, *p* = 0.38 respectively) reported by Gorczyca [30].

Disease activity and health-related quality of life in relation to dietary patterns and supplements in JIA.

Gorczyca and colleagues [30] in a case–control study with 110 participants found that omega 3 and omega 6 PUFAs negatively correlated with inflammatory markers ESR and CRP (*p* < 0.05).

According to Çakır et al. [37], serum 25(OH) vitamin D levels of the 100 healthy subjects were significantly higher compared to the vitamin D levels of the patient group with 117 participants (*p* < 0.01). There was no statistically significant correlation between vitamin D levels and the number of joints with active arthritis (r = 0.1, *p* = 0.4). In a retrospective study by Çomak et al. [38] with 47 participants, no significant difference was found between disease activity and 25(OH) D levels in children who consumed vitamin D supplements compared those who did not (*p* = 0.053 and *p* = 0.021, respectively), whereas there was a significant negative correlation between 25(OH) D levels and disease activity as described by JADAS-27(*p* = 0.01, r = -0.37, d = -0.7965). Pelajo et al. [9] in a cross-sectional study with 154 participants reported serum 25(OH) D levels were not associated with JADAS-27 (beta coefficient = 0.002; 95%CI = -0.1, 0.1; *p* = 0.97), although in new-onset patients there was a negative correlation (r = -029 and d = -0.60) and positive correlation with long term JIA patients (r = 0.06, d = 0.12). Shevchenko et al. [25] in a case control study with 84 participants found no relationship between vitamin D and disease activity (r = -0.11, *p* > 0.05). In another cross-sectional study by Bouaddi et al. [10] with 40 participants Serum 25(OH)D levels were associated with disease activity (*p* = 0.04, β: − 3.87) (Fig. 2).

### Results from interventional studies

Disease activity and health-related quality of life in relation to dietary patterns and supplements in JIA.

Gheita and colleagues [22] in a controlled trial study with 47 participants, evaluated the effect of dietary supplements of Ɯ-3 PUFA for 12 weeks intervention, with an improvement in the mean active joint count (*p* < 0.001) and Juvenile Arthritis Disease Activity Score 27(JADAS-27) (*p* < 0.001) in JIA group. However, there is a high risk of bias in the study based on the Cochrane tool for randomised trials. Similarly, Yareman et al. [23] in a randomised controlled trial with 68 participants, demonstrated the ω-3 PUFA over three months of intervention had a positive effect on the comprehensive treatment of inflammatory diseases (*p* = 0.05) even though there are some concerns in the study.

According to Tang et al. [19] disease activity did not improve after receiving vitamin D supplements for 24 weeks in 18 children with JIA (*P* > 0.05). Berntson et al. [24, 40, 41], in one case and one pilot study with one participant, and one exploratory study with seven participants, reported that inflammation, pain and disease activity improved after full nutrition with EEN (which contains protein and what is required for complete nutrition). Furthermore, EEN reduced inflammatory proteins such as MCP-2, MCP-3 and MCP-4 (monocyte chemoattractant protein), resulting in clinical improvement in 7 patients with JIA. Golini, in a clinical trial study with 16 participants, showed that Kre-Celazine nutritional supplement (composed of a proprietary alkali buffered, creatine, monohydrate and fatty acids mixture) have improved pain and decreased inflammatory indexes (ESR and CRP). [21].

Dilandro et al. [12] in a control trial study including 22 participants showed biscuit supplementation enriched with an iron over the 4-month period, decreased the inflammatory indexes particularly ESR (*p* = 0.002).

## Discussion

To our knowledge, this is the first systematic review and meta-analysis to look at the evidence for the impact of diet interventions, supplements and other nutritional factors in children and young people with JIA. Importantly there were no studies assessing particular dietary patterns on symptoms in JIA.

Overall, based on the findings from interventional non-controlled studies, there was some evidence that dietary supplements Kre-Celazine and full nutrition by EEN may have potential for a beneficial effect on symptoms such as pain, inflammatory markers and disease activity in patients with JIA. However, this should be interpreted with caution as controlled studies with larger sample sizes in children are required to confirm the results. Although, observations from the research in adults should be considered where an association between disease severity in arthritis and food intake has been demonstrated [42] and the role of the gastrointestinal mucosal immune system in the pathogenesis of rheumatoid arthritis has been strongly evidenced [42].

We further observed that in two controlled trial and one case control studies, ω-3 FAs reduced pain, joint tenderness, morning stiffness, inflammatory markers, and over time, the NSAIDs dose needed by JIA patients in addition to improvement of the JADAS-27, CHAQ scores and improved immune system [20, 21, 26]. This is similar to most studies on ω-3 FAs in RA adults. However, findings in those with JIA should be interpreted with caution, as studies covered a relatively small group of patients with a high dropout rate and thus prone to bias. It may be beneficial to do high-quality research with a larger sample size to clarify the efficacy of ω-3 FAs in children and young people with JIA.

Research is conflicting in regards to the impact of vitamin D on rheumatic symptoms in both children and adults. With respect to vitamin D levels in children and young adults with JIA, two out of six studies [10, 38] reported a significant negative correlation between disease activity and 25(OH) vitamin D levels independent of age, gender, JIA subtype, disease duration, medications and BMI. In addition, patients with higher disease activity levels have vitamin D deficiency. These results are consistent with similar studies in adults [43–46]. Other studies in children with JIA have yielded contradictory results [9, 19, 37, 45] and did not observe a relationship between 25(OH) D level and disease activity, which is consistent with the findings by Braun-Moscovici et al. [47] in adults. Despite being of fair to high quality, future studies with larger sample sizes that consider and adapt analysis for other contributing factors, and importantly studies in new-onset and long-term JIA patients are required to confirm the results of the above studies.

There was some evidence from observational studies for the relationship of dietary supplements and blood markers of vitamins and minerals to growth rate, weight and disease activity and life quality with overall poorer diet relating to worse health. However, the data was variable and difficult to interpret in relation to today's cohort of children due to the study designs, studies span a significant time, through which there have been substantial developments in the treatment of JIA specifically the widespread use of biologic medications. Well-designed interventional studies required.

Regarding nutrient status [35], disease activity and subtype of the disease, there are no significant differences when compared to plasma copper and zinc levels. Bacon et al. [31] confirmed the results of this study. However, children with JIA as well as healthy children presented lower RDI of copper and zinc [35]. For those with JIA, this may signify a greater deficit, as there may be an increase in the requirements caused by the inflammatory process [26]. On the other hand, the study by Stark reported that [20] in taking calcium from food in children with JIA was increased by behavioural change interventions, while previous intervention studies on calcium have used calcium supplements, calcium-enriched foods, to achieve increases in calcium intake.

Considering anthropometric measurements, one study [32] found that children with JIA had more total body fat compared with healthy children. These findings agreed with studies by Knops [48] and Summers [49]. These results are inconsistent with the studies by Haugen, Lofthouse and Mortensen (26, 34, 36) who showed that weight and height were reduced in JIA patients, although differences in measurements between the JIA patients and controls were not statistically significant. Lack of significance could be due to the small number of participants in the study; studies with a greater sample size are required to assess the anthropometric measures in JIA patients.

To its strength, a comprehensive and inclusive search strategy were used and composed with input from a literature-searching specialist and implemented across multiple databases.

The main limitation of this review is bias due to heterogeneity, in which individual studies varied in their inclusion/exclusion of specific JIA subtypes. Due to resource constraints, only studies in English were included; however, we felt it unlikely that this led to omission of major relevant studies.

## Conclusion

To our knowledge and based on the results of this systematic review and meta-analysis, there is no study to date that assesses the relationship between dietary intake, symptoms and health-related quality of life in children and young people with JIA. The findings of this systematic review indicate that supplements such as Ɯ-3 FAs and full nutrition by EEN may show potential for improving JIA symptoms, while other supplements, including vitamin D, were found not to be associated with improvement of JIA symptoms nor a better quality of life. In addition, the reporting of weight and height, in some studies indicates children with arthritis had a significantly lower weight than healthy controls, while in other studies, obesity was reported to be greater in children and young people with JIA. Different countries with different nutritional norms, routines and supplementation might justify the differences. It is not clearly understood to what extent diet causes low levels of different nutrient components. It could as well be the disease itself, caused by aberrations in the gastrointestinal function, the possibility to absorb nutritional components, or caused by medication etc. Well-designed, carefully measured interventional studies of dietary patterns in combination with important contributing factors such as medication and life style behaviours including physical activity are required to determine the impact of diet in improving symptoms and growth patterns in children and young people with JIA, with an aim to improve the quality of their life.

## Supplementary Information

**Additional file 1.**

**Additional file 2.**

**Additional file 3: Table 1. **Characteristics of the selected studies (trials, exploratory, case and pilot studies).

**Additional file 4: Table 2. **Characteristics of the selected studies (case-control, cross-sectional, cross-sectional with control and cross-sectional cohort).

## Declarations

### Availability of data and materials

All data generated or analysed during this study are included in this published article [and its supplementary information files].
