# Supplementary material for: An investigation into the relationship between nutritional status, dietary intake, symptoms and health-related quality of life in children and young people with juvenile idiopathic arthritis: a systematic review and meta-analysis
Source: BMC Pediatr. 2023 Jan 2;23:3. doi: 10.1186/s12887-022-03810-4 (PMC9806873; doi:10.1186/s12887-022-03810-4)
Supplement: Supplementary file 1 — Additional file 1. [file 12887_2022_3810_MOESM1_ESM.docx]

Appendix 1: search terms

We used the following search criteria: “Juvenile idiopathic arthritis” or JIA or RA or Arthritis* or “rheumatoid arthritis” and nutr* or food* or diet* or Eat*or supplement* or vitamin* and “Quality of life” or life style* or “lifestyle” or “life-style” or “QoL” or “HRQoL” or life qualit* or “health related quality of life” or value of life or health- related quality of life or health status or wellbeing or well being or well-being or wellness or mental health. “Juvenile idiopathic arthritis” or JIA or RA or Arthritis* or “rheumatoid arthritis” and nutr* or food* or diet* or Eat*or supplement* or vitamin* and “Weight” or “obesity” or “adiposity” or “LDL” or “low density lipoprotein” or “HDL” or “high density lipoprotein” or “Blood pressure” or “fitness” or “health” or “health behaviour” or “health behavior”. “Juvenile idiopathic arthritis” or JIA or RA or Arthritis* or “rheumatoid arthritis” and nutr* or food* or diet* or Eat*or supplement* or vitamin* and “Pain” or “joint mobility” or “walking difficulty” or “Sleep impairment” or “fever” or “fatigue” or “morning stiffness” or “swelling” or “inflammation” or “anxiety” or “depression” or “Negative feelings”. “Juvenile idiopathic arthritis” or JIA or RA or Arthritis* or “rheumatoid arthritis” and nutr* or food* or diet* or Eat*or supplement* or vitamin* and Inflammation* or “diagnosis”. “Juvenile idiopathic arthritis” or JIA or RA or Arthritis* or “rheumatoid arthritis” and nutr* or food* or diet* or Eat*or supplement* or vitamin* and “Quality of life” or life style* or “lifestyle” or “life-style” or “QoL” or “HRQoL” or life qualit* or “health related quality of life” or value of life or health- related quality of life or health status or wellbeing or well being or well-being or wellness or mental health and “Weight” or “obesity” or “adiposity” or “LDL” or “low density lipoprotein” or “HDL” or “high density lipoprotein” or “Blood pressure” or “fitness” or “health” or “health behaviour” or “health behavior” and “Pain” or “joint mobility” or “walking difficulty” or “Sleep impairment” or “fever” or “fatigue” or “morning stiffness” or “swelling” or “inflammation” or “anxiety” or “depression” or “Negative feelings” and Inflammation* or “diagnosis”.
