# Supplementary material for: An investigation into the relationship between nutritional status, dietary intake, symptoms and health-related quality of life in children and young people with juvenile idiopathic arthritis: a systematic review and meta-analysis
Source: BMC Pediatr. 2023 Jan 2;23:3. doi: 10.1186/s12887-022-03810-4 (PMC9806873; doi:10.1186/s12887-022-03810-4)
Supplement: Supplementary file 2 — Additional file 2. [file 12887_2022_3810_MOESM2_ESM.docx]

**Table 2:** characteristics of the selected studies (case-control, cross-sectional, cross-sectional with control and cross-sectional cohort)

| **Author**  **year** | **Study method** | **location of the study** | **age/gender** | **subtypes of arthritis** | **primary aim** | **secondary aim** | **measurements** | **values** | **primary outcome** | **secondary outcomes** |
| --- | --- | --- | --- | --- | --- | --- | --- | --- | --- | --- |
| **2019**  **Shevchenko N., Khadzhynova Y.** | Case-control | Ukraine | Total:69  F: 45  M**:** 24  Average age:10.9 ±4.8 | Oligo A: 25  Poly A: 36  undifferentiated A: 10 | Assess the possible relationship  between the level of vitamin D, main features of patients (age,  gender) and clinical characteristics of disease (duration and activity of the disease, number of joints involved in JIA. | N/A | Lab test:   - serum vit D levels - ESR   Disease activity:  Juvenile arthritis disease activity score (JADAS27). | number of active  joints :   - Oligo A: 2.1±1.2. - Poly A: 4.7 ± 2.6. - Undifferentiated A: 1.2± 0.7.   JADAS27  • Oligo A: 16.6± 6.6.  • Poly A: 21.8±10.7.  • Undifferentiated A: 10.8±5.6.  level of 25(OH)D:  • Oligo A: 24.7 ±7.2.  • Poly A: 22.3 ± 7.8.  • Undifferentiated A: 18.9 ±8.2. | - Vitamin D levels in healthy children was significantly higher (p>0.05). - No correlation between vitamin D status and duration of disease (r – 0, 12; P>0, 05). - No correlation between vitamin D status and activity of disease (r-0, 11; P>0, 05). - No correlation between vitamin D status and number of active joints( r-0,05; P>0,05) - No correlation between vitamin D status and number of injured joints (r-0, 14; P>0, 05). | N/A |
| **2014**  **Bouaddi et al** | Cross-sectional | Morocco | Total:40  F:18  M:22  Age:11 ± 4.23 | Systemic-onset A: n= 11  OA: n= 9  PA(RF-) n= 1  PA(RF+) n= 17  ERA n=1  Psoriatic A: n=1 | Examine the association between  serum levels of 25(OH)D and disease activity | N/A | Lab test:  • ESR  •CRP    CHAQ  Disease activity score 28(DAS 28)  Anthropometric measurements: BMI | - No of ender joints :3 (p= 0.02) - No of Swollen joints :1 (p= 0.2) - ESR (mm/h)1 34.5 [25–55](p= 0.05) - CRP (mg/l)1 21.5 [11–41] - Patient global assessment (cm)1 25 [10–40] - DAS 28: 4.84 ± 1.27. (p= 0.04) | Serum 25(OH) D were associated with DAS28 (p = 0.04, β: −3.87, CI: (−7.67,-0.07).  serum 25(OH)D levels were associated with the following disease activity components:   - ESR (p = 0.05, β: −0.14, CI: (−0.28,0.004)), - Tender joints (p = 0.02, β: −0.79, CI (−1.47,-0.10)). - Patient global health (p = 0.04, β: −0.17, CI: (−0.35,-0.004).   No association between 25(OH)D and  DAS28:( β−3.87,CI:−7.67, −0.07,p= 0.04)  ESR:( β= −0.14 ,CI−0.28, 0.004,p= 0.05)  Tender joints: (β= −0.79 CI: −1.47, −0.10,p=0.02)  Patient global health: (β= −0.17, CI: −0.35, −0.004,p=0.04). | N/A |
| **2014**  **M-M Grönlund et al.** | cross-sectional cohort | Finland | Total:80  JIA:40  Control:40  JIA:3.2-10  Mean age:6.4  F:21  M:19  Control: 5.0–7.1  Mean age:6  F:21  M:19 | Persistent OA n=20  Extended OA n=5  PA(RF-) n=14    ERA n=1 | Assess the effect of JIA, its subtypes and disease activity on anthropometric measurements, body composition, and nutritional parameters. | N/A | Anthropometric measurements :  •Height  •Weight  •SFT  •body fat  •MAC  •PMC  7-day food diary  lab test:  •ESR  •CRP  •hsCPR  •IL-6  •BC  •Alb,  •25(OH) D  •folate  •ferritin  •LDL  •transferrin receptor  •( IL)-6  CHAQ  Clinical assessment:  •Time of onset of JIA  •Current medication  •Presence or absence of uveitis.  •Number of active joints  •Disease activity  VAS(0-100) | • No. of active joints:0(0-5)  • No. of joints with limited range of motion: 0(0-5)  • Physician VAS score:9(0-40)  • Patient/parent VAS score:5(5-40)  • 25(OH)D :67.2(17.6)  Serum calcium :  • JIA:2.3(0.059)  • Control:66.1 (13.0  • P-value:0.75  Height/mean, SD:  • JIA:-0.10(0.93)  • Control:0.04(0.99)  • P-value:0.54  Body fat/Mean, SD:  • JIA:17.1(4.7)  • Control:15.4(4.3)  • P-value:0.11 | Positive correlation between CHAQ and number of active joints with the proportion of body fat (R 0.48, p 0.002 and R 0.34, p 0.034, respectively)  Weight (kg), mean (SD):   - JIA: 23.6(6.2) - control: 21.0(3.6) - p values: 0.029   W(cm),mean(SD):  JIA:55.9(4.9), Control:52.3(4.3)p-values:<0.0001  BSF thickness:   - JIA: 6.2(2.3) - Control: 5.3(1.7) - p-values: 0.035   Erythrocyte folate level   - JIA:792(251) - control:590(119) - p-values: 0.0025 | N/A |
| **2007**  **Marcela Gonçalves et al** | Cross-sectional study  with control group | Brazil | Total:103  JIA:51  F:37, age  M:14  age range: 2.3–17  Mean age:11.3  Control: 52  F: 42  M:10  age range 3–18  Mean age: 12.5 | Oligo A: 22  PA: 17  Systemic A: 12 | Evaluate Plasma Hcy in patients with JIA and its correlation with MTX, folate and B12 and hyperlipidaemia. | Relation between HCy level and MTX, disease onset and clinical and laboratory parameters. | **Lab test:**   - ESR - CRP - Plasma Hcy - Serum folate - B12 - TG - TC - LDL - HDL - VLDL - Cr   **Disease activity**:   - Active JIA: one or more joints with arthritis at the time of the study. | Not mentioned | **Hcy:**   - Pt:9.27 (3.16) - Control: 8.92 (2.42) - p-value: 0.615   **Vitamin B12**   - Pt:552(315.5) - Control: 484.5 (246.6) - p-value: 0.341   **Folate**   - Pt: 11.25 (4.72) - Control: 6.66 (2.27) - p-value: < 0.001   **VLDL**   - Pt:15.43(5.19) Control:20.12 (11.01) - p-value: 0.014   **HDL**   - Pt:39.0 (7.67) - Control:43.58 (9.21) - p-value: 0.007   **LDL**   - Pt:94.75 (20.41) - Control:89.81 (19.15) - p-value:0.208   **TG**   - Pt:71.24 (23.33) - Control: 93.98 (41.17 - p-value 0.001 | No significant differences in relation to the onset and course types of disease and MTX.  No significant correlation among Hcy plasma concentration or clinical and laboratory parameters in JIA patients. |
| **2017**  **Daiva Gorczyca et al.** | case-control | Poland | Total:108  JIA:66  F:50  M:16  JIA age:1.5-18(8.6)  Control:42  F:29  M:13  Control age: 7-17.8(8.8) | oligo-A: 38  PA: 18 | Association between  of n-3 and n-6 PUFAs, serum  profiles, and immune and inflammatory markers in  JIA in relation to onset, activity, and duration of the disease. | N/A | **clinical  examination:**   - Age at disease                onset   - Age at consent - Duration of disease - Number of active/swollen joints - Number of joints with limitation of motion - Presence of uveitis - Duration of morning stiffness   **Anthropometric measurements:**   - Weight - Height - BMI   **lab test:**   - RF - anti-CCP antibodies - ANA positivity - ESR - CBC - CRP - n-6 PUFAs - AA - n-3 PUFAs - ALA - EPA - IL-6 - IL-10 - IL-17A - DHA   **The dietary PUFA intake:**  7-day dietary record. | **BW median:**   - 25.05(10.10–102.00) - 28.95(13.90–70.00) - p value:0.26   **BH median:**   - 127 (77-175) - 128 (100-196) - p value:0.38   **BMI median:**   - 16.45 (12.01–34.40) - 17.05 (13.76–30.00) - p value:0.46 | Significantly higher total levels of n-6 PUFA and LA in inactive JIA compared to active JIA (p =0.018, p =0.024, respectively).  Significantly lower levels of AA in active JIA and with short-lasting disease than the healthy control (p =0.003, p =0.012, p = 0.002, respectively).  Significantly higher levels of  ALA in the poly-JIA and oligo-JIA than in healthy controls (p = 0.042, p =0.022, respectively).   Significantly lower levels of DHA in active and  short-lasting JIA in comparison to the healthy control group  (p = 0.015,p = 0.024,respectively).  Lower levels of DH in active patients than in inactive patients (p =0.028).  Significant higher levels of  IL-10 in JIA with active disease, oligo- poly JIA, short-lasting and long-lasting disease in compare on to the healthy control group (p = 0.008, p = 0.008, p = 0.047, p = 0.018, p= 0.036, p = 0.020, respectively).  Negative correlation between  n-3 and n-6 PUFAs with CRP and ESR (p < 0.05)  Positive correlation between n-3 and n-6 PUFAs and platelet count (p< 0.05). | N/A |
| **1989**  **Carol J. Henderson and Daniel J. Lovell** | cross-sectional | USA | Total:28  Age range: 5.1-16.5  Mean age: 10.6  F:54٪  M:46 % | Systemic: 7  PAr:11  Pauci-A:10 | Assess the Protein-Energy Malnutrition in Children and Adolescents with JRA. | NA | **Anthropometric measurement**:   - Weight - Height - TSF - SSF - TAC - AMC - AMA   **Disease severity:**     - Number of swollen joints - Number of tender joints   **Lab test:**   - Albumin - Prealbumin - RBP - ESR | **<5th  percentile**  **for age**  **and sex-matched for:**   - Height - Weight - TSF - AC - SSF - AMC - AMA   **Adjusted weight-for-height index body weight for current height:**   - <0.80   **<3.5 g/d age-adjusted**  **<5th percentile for age-adjusted norms**   - Serum albumin - Serum prealbumin - Serum retinol-binding protein | **Referred (n = 10)**   - Protein-energy malnourished:7 - Some Abnormalities:3 - Not at risk: none   **Inpatient (n = 8)**   - Protein-energy malnourished:7 - Some Abnormalities:3 - Not at risk: none   **Inpatient (n = 8)**   - Protein-energy malnourished:6 - Some Abnormalities:2 - Not at risk: none   **Outpatient (n = 2)**   - Protein-energy malnourished:1 - Some Abnormalities:1 - Not at risk: none   **Screened (n = 18)**   - Protein-energy malnourished:3 - Some Abnormalities:5 - Not at risk: 10 | N/A |
| **2003**  **Silverio Amancio et al.** | cross-sectional with control group | Brazil | Total:64  JRA:41  M:20  F: 21  Mean age: 11.3  Age range:  3.3 – 17.8 y  Control:23  F:13  M:23  Mean age: 9.9  Age range: 4.7 – 17.2 y | Active phase:  Pauci-A:8  PA:13  Non Active phase:  Pauci-A :11  PA:9 | Evaluate the copper and zinc intake and serum levels in patients with JRA (Pauci-A and PA), the disease activity and duration, the number of inflamed joints and the use of  Corticosteroids therapy. | N/A | **Dietary evaluation:**   - four-consecutive-days food register(   The copper and zinc validated and standardized Virtual Nutri-USP (Philippi et al, 1996) software.  **Lab test:**   - Copper - Zinc | **Copper and zinc relation to :**  Activity (n =21)  No activity (n =20)  **Copper:**   - 156.9±39.6 - 129.5±25.1 - (P =0.012)   **Zinc:**   - 92.1±16.6 - 95.5±12.3 - P =0.469   Pauci-A(n =19)  PA (n =22)  **Copper:**   - 136.8±37.6 - 149.3±33.8 - P =0.270   **Zinc:**   - 94.4±10.5 - 93.1±17.6 - P =0.774   Corticosteroid therapy  N=8  Non  Corticosteroid therapy  N=5  **Copper:**   - 63.1±36.7 - 159.0±26.3 - P =0.832   **Zinc:**   - 89.3±21.1 - 88.0±15.2 - P =0.902   **Duration of disease**  **PA type(n=41)**   - Copper:_.011 - p:0.946 - Zinc: 0.214 - p=0.178   Number of inflamed joints(n=21)  Polyarticular type   - Copper: 0.494 - p=0.001 - Zinc: 70.297 - p= 0.059   **copper and zinc intake, determined by a 4-day food register in JRA patients with and without disease activity:**  **Activity(n=21):**   - Copper: 1.15±0.57 (0.6 – 3.2) - Zinc: 8.42±3.7   (1.1 – 16.3)  **No Activity** **(n=20):**   - Copper: 1.24±0.64   (0.6 – 3.2)   - Zinc: 8.04±2.5   (3.1 – 12.9) | - Higher copper levels in male JRA than male in the control group: p=0.004 - Statistically significant relationships between disease activity and the number of inflamed joints with copper levels p=0.012 and p=0.001, respectively. | No statically significant difference between JRA characteristics, copper, and zinc. |
| **1990**  **Bacon et al.** | case-control | USA | Total:43  JIA:34  Control: 9  Age:3-17  JIA:  Systemic :  F:4  M:4  Mean age:8.2  PA:  Mean age:11.4  F:10  M:4  Pauci-A  Mean age:10.2  F:7  M:5  Control:  Mean age: 8.9  F:4  M:5 | Systemic  PA  Pauci-A | Examine the relations among dietary  habits, nutritional status, and physical  Growth of JRA. | N/A | **Lab test:**   - Albumin - Transferrin - Prealbumin - RBP - Somatomedin C - Vitamins A, C,E - Ca - P - Iron - Zinc - Copper - Selenium   **Anthropometric Measurements:**     - Weight - Height - AMA - BMI   **3 day dietary intak**e | **Height for Age**: **mean(SD)**   - Systemic: 32.3 (38.2) - PA: 30.1 (22.6) - Pauci-A: 46.3 (3 1.8) - Controls: 67.5 (28.1)   **Weight for Height:** **mean(SD)**   - Systemic: 84.0 (17.8) - PA: 43.6 (35.2) - Pauciarti-A: 55.9 (28.7) - Controls: 62.6 (39.4)   **Uppe AMA** **mean(SD):**   - Systemic: 29.4 (25.0) - PA: 27.9 (36.9) - Pauci-A: 32.8 (28.3) - Controls: 41.9 (31.2)   **Biochemical Indices of Nutritional Status, mean(SD):**  **(Significantly different from control:* p< .05,**p≤ .O1)**  **A retinol:**   - Systemic: 0.32 (O.1O) ** - PA: 0.29(0.07)** - Pauci-A: 0.36 (0.07)* - Controls: 0.44 (0.17)   **C ascorbic acid (wg/mL):**   - Systemic: 3.3 (1.6)* - PA: 4.8 (l.S)** - Pauci-a: 7.0 (1.7) - Controls: 6.6 (1.8)   **E a-tocopherol (Fg/mL):**   - Systemic: 8.5 (2.1)* - PA: 7.4 (3.2) - Pauci-A 9.0 (3.7)* - Controls: 6.1 (1.0)   **Zinc (Mg/dL):**   - Systemic: 80.5 (13)* - PA: 85.9 (15)* - Pauci-A: 87.5 (12)* - Controls: 98.3 (16)   **Copper (Fg/dL):**   - Systemic: 139 (40)** - PA: 133 (29)** - Pauci-A: 122 (33) - Controls: 100 (13) | A significant abnormality was seen in nutritional status in systemic and PA JRA.   - Zinc: p ≤05 - Vit A, C and copper: p≤.O1 - No significant correlation between diet, nutritional status and growth in any of the three types of JRA was seen. | N/A |
| **1990**  **A. L. MORTENSEN et al.** | cross-sectional | Australia | Total:38  Pauci-A  Age(years):7.4±4.4  M:3  F:10  Systemic:  Age(years):6.8±4.4  M:5  F:5  PA:  Age(years):9.1±4.3  M:5  F:10 | Pauci-A:13  PA:15  Systemic onset:10 | Assess the nutritional status and dietary adequacy in children with JCA. | N/A | **Anthropometric measurements:**   - Weight - Height - MUAM - MUAF - MTSM   **Dietary analyses:**   - 7-day record   **lab test:**   - ESR - CRP | **Anthropometric variables of groups by disease type:**  **Z  height score:**   - Pauci-A: -0.16±1.0 - Systemic:1.1±1.1 - PA: 0.75+0.96   **Z weight score:**   - Pauci-A: +0.18 ±1.19 - Systemic: -0.39± 1.2 - PA: 1.07±1.0   **Percent ideal weight for height age:**   - Pauci-A: 105±10 - Systemic: 109±16 - PA: 94±11   **MUAM<5th percentile:**   - Pauci-a: 01:11:00 - Systemic: 216 - PA: 04:13:00   **MUAF<5th percentile:**   - Pauci-A: 03:11:00 - Systemic: 2.6 - PA: 6.13 | - Mean energy intakes were significantly below the RDI in the systemic (P = 0.01) and PA(P=0.001) groups. - Mean intakes of calcium and zinc were below the RDI of 100% in the PA group(P = 0.001) - The mean intakes for iron, thiamine, niacin equivalents, riboflavin, Vitamins C and A were all above the RDI in each group. | N/A |
| **2016**  **Aydilek Dağdeviren-Çakır et al.** | cross-sectional case-control | Turkey | Total:217  JIA  active disease  :n=64  JIA in remission: n=53  Healthy: n=100  Age:  JIA active disease: 9.7±4.3  JIA in remission: 9.8±4.3  Healthy: 9.9±4.1  F:41 (active disease)  M:23 (active disease)  F: 35(remission)  M:18(remission) | Systemic :13  PA :34  ERA:5  OA :61 | Determine the prevalence of vitamin D deficiency and/or insufficiency and investigate the relationship between vitamin D and disease activity in patients with JIA and FMF. | Determine the relationship between:  ca, p and alk in healthy group and JIA.  Vit D levels and ESR and CRP.  Vit D levels and drug regimen.  between activation and remission periods in terms of the medications used. | **Lab test:**   - ESR - CRP - WBC - 25(OH) vitamin D - Ca - P - ALK   **Disease activity:**     - JADAS-27 | **Vitamin D Levels:**  JIA(Activation period)   - Mean Vit D: 18.9±11                16.5(4.6-45)  JIA(Remission period)  (n=53)   - Mean Vit D:                   18.6±9.2                   17.05(5.45-55)  Control Group   - 26.71±10.54 - 27.2(6.8-61.3)   **JIA(Activation period):**   - ESR: Mean (SD): 46.6±30.5 - CRP:   Mean(SD):3.41±3.54  **JIA(Remission period):**   - ESR: Mean(SD): 18.1±12.6 - CRP**:** Mean(SD):0.78±1.54 | - No significant difference between vitamin D levels of the patients with JIA during activation and remission periods. - Significantly higher levels of Serum 25(OH) vitamin D in healthy subjects compared to the patient groups (p<0.01). - No statistically significant correlation between vitamin D levels and the number of joints with active arthritis (r=0.1, p=0.4) and physician and family VAS assessments (r=0.03/p=0.77, r=0.03/p=0.78 respectively). | No difference between activation and remission periods in terms of the medications used.)    Normal calcium, p, Alk leves both in the healthy control group and in the patient group.  No statistically significant correlation between the vitamin D and ESR and CRP s (r=0.2, p=008;r=0.08, p=0.5, respectively) in the patients with JIA.  No relationship between the drug regimens [steroids, disease-modifying anti rheumatic drug (methotrexate,sulfasalazine, leflunomide)  TNF-alpha antagonist, new generation biologic drugs (IL-1 antagonist, IL-6antagonist, T-cell antagonist)] and vitamin D levels could be shown (p=077,p=0.6, p=0.1, p=026 respectively). |
| **2014**  **Elif Çomak et al.** | Retrospective study | Turkey | Participants:47  F:29  M:18  Mean age:  9.3±3.9 | OA: 20  PA(RF-): 6  PA(RF+): 1  Systemic-onset: 13  ERA:6  Psoriatic: 1 | Evaluate vitamin D status in patients with JIA | Examine the association between serum levels of 25(OH)D and disease activity in JIA. | **Lab test:**   - ESR - serum 25(OH) D levels - WBC - CRP   **anthropometric measurement:**   - BMI - Weight - Height   **Disease activity:**   - JADAS-27 | **Mean serum 25(OH) D:**     - 17.7±11.6 ng/ml.   **Vitamin D insufficiency:**     - 15-20 ng/ml (9 (19.1%)   V**itamin D deficiency:**     - <15 ng/ml ( 25 (53.2%)   **Adequate vitamin D levels:**   - (13 (27.7%) | - No significant difference between disease activity in and 25(OH) D levels of the children who used vitamin D supplements and those who did not (p=0.053 and p=0.021, respectively). - There was a significant negative correlation between 25(OH)D levels and disease activity(p=0.01, r=-0.37) - There was a significant negative correlation between 25(OH) D levels and physician VAS, parent VAS and joint count (p=0.001, p=0.001, p=0.02, respectively). - The mean JADAS-27 scores were 10.9±5.7 and 6.6 ± 4.5 in patients with 25(OH)D levels <15 ng/ml(Vit D deficiency) and 25(OH)D levels >15 ng/ml (p=0.003) | No association was found between 25(OH)D levels and age, gender, JIA subtype, disease duration, JIA medications, inflammatory parameters (WBC, ESR and CRP), vitamin D supplements, use of prednisolone or BMI: ( p>0.05)  No difference was found between the serum 25(OH)D levels of the 11 children who used prednisolone and the levels of those who did not (17.9±6.4 and 17.7±1.3 ng/ml, p=0.213).  No significant correlation between disease activity and cumulative dosage of prednisolone: p=0.06, r=0.39. |
| **2012**  **Michelle Cavalcante Caetano et al** | Cross-section controlled study | Brazil | Total:77  JIA:42  F:42  M:0  JIA age:6-19  Mean age:13  Control:35  Control age:5.2-19  Mean age: 11 | PA: 23  OA: 17  Systemic: 2 | Evaluate the BC of JIA girls compared with healthy control and the associations between total fat mass and disease status and treatment. | N/A | **Clinical signs:**   - Fever - Rash - Pain - Stiffness - Number of the active and limited joints.   **Lab parameters:**   - ESR - CRP   **Anthropometric Measurements:**   - Weight - Height   **Nutritional status:**   - Evaluated according to the WHO standards   **Body composition**   - DXA | **Z-BMI**   - JIA: 0.17 (−3.8–+2.9) - Control: −0.48 (−1.4–+1.00) - P: 0.034   **Z-H**   - JIA: −0.33 (−4.8–+1.2) - Control: −0.59 (−1.9–+1.5) - P: 0.834   **TBF:**   - JIA: 26.5 (12.7–50) - Control: 16.4 (6.4–37.7) - P: 0.001   **TFM:**   - JIA: 4.52 (0.5–15.7) - Control: 2.32 (0.6–9.7) - P: 0.011   **FMI**   - JIA: 4.83 (1.4–14.4) - Control: 2.23 (0.7–8.5) - P: <0.001   **LMI**   - JIA: 13.45 (1.0–15.6) - Control: 12.45 (9.8–16.7) - P: 0.212 | A significantly greater percentage of total body fat (p=0.001) truncal fat (p=0.011) and FM (p<0.001) in JIA girls compared with controls but did not find a difference in LMI. | N/A |
| **1992**  **M. A. Haugen et al.** | case-control | Norway | Total:32  JCA:15  Control:17  JCA:  F:12  M:3   Age in Pauci-A :11.6-13.0, Mean age:12.5  Age in PA: 11. 0-14.5   Mean age:13.4  Control:  F:11  M:6  Age: 11.5-13. 8  Mean age:13.2 | Pauci-A JCA  PA JCA | Relationship between nutritional status and disease activity to dietary Intake in JCA | N/A | **Lab test:**   - Alb - TIBC - RBP - B12 - Zinc - Copper - Selenium - Hb - ESR   **Anthropometric measurements :**   - Height - Weight - UAC - TSF - UAMA   **7-day record food**  **Disease activity:**  on a 6-point scale   - 0 = no disease activity, - 1 = low disease - activity - 2 = moderate disease activity - 3 = high disease activity - 4 = Severe disease activity - and - 5 = very severe disease activity - ESR - CRP | **Disease activity(none):**   - Pauci-A: 0 - PA: 0   **Disease activity(low):**   - Pauci-A: 4 - PA: 0   **Disease activity(moderate):**   - Pauci-A: 3 - PA: 3   **Disease activity(high):**   - Pauci-A: 0 - PA: 4   **Disease activity(severe):**   - Pauci-A: 0 - PA: 1   **Disease activity(very severe):**   - Pauci-A: 0 - PA: 0   **ESR:(median-range):**   - Pauci-A: 8(3-13) - PA: 17(8-54) - Control: 5(2-12)   **Height median,**  **95% c.i.**   - Pauci-A: 155(142-183) - PA: 152 (140-158) - Control: 157(153-161)   **Weight median,**  **95% c.i.**   - Pauci-A: 54.0(39.3-60.3) - PA: 36.8 (30.0-47.9) - Control: 46.0(44.8-49.8)   **Energy(kcal/day)**   - Pauci-A: 1695 (1315-2410) - PA:   1845(1475-2057)   - Control:2000(1390-2245) | - A significantly lower UAMA value in the PA group than the Pauci-A and the control group was seen (both p<0.01). - Significantly lower weight in  PA group a than both the healthy controls (p=0.01)and pauci-A(p = 0.02). - Reduced concentration of   Iron and zinc in the PA group compared to Pauci-A (p=0.02) and healthy controls (both p<0.01).   - Significantly higher disease activity and ESR in the PA group with the pauci-A  (p= 0.02 and p<0.01 respectively). - Positive correlation seen between ESR and the disease activity t (r = 0.54, p < 0.01). - Higher energy intake per kg body weight in the PA group than in the Pauci-a group (p=0.04). - Reduced Ca intake was in the PA group compared to that of the control group (p= 0.05). | N/A |
| **2000**  **R.P. Harper et al** | case-control | USA | Total:78  Age:6-18  Mean age:2.1±3.5  Control:34  JRA:44  JRA Positive for TMD: 24  Female: 18  Male:6  JRA Negative for TMD: 20  Female: 14  Male: 6 | JRA with TMD  JRA without TMD | Examined the relationship between  self-report measures of pain and dysfunction and measures of chewing  Performance in JRA and healthy controls. | Assess the incidence of TMD involvement by gender | **TMD assessment:**     - Range of mandibular motion - TMJ sounds - Craniofacial muscle pain - Chewing pattern   **Qol:**   - Self-report   **pain:**     - Visual and analogue(0-10) | **Self-Report of Pain and Dysfunction: Correlation between Visual and Analog Scales(before chewing task)Baseline**  **•P=0.001, ••P<0.01**  **JRA with TMD**   - Jaw pain: 0.92• - Ability to chew: 0.76• - Quality of life: 0.97•   **JRA without TMD**   - Jaw pain: N/A - Ability to chew: N/A - Quality of life: 0.72••   **Control group:**   - Jaw pain: 0.55• - Ability to chew: 0.82• - Quality of life: -   **Differences among Groups for Analog Self-Report Measures( Before Chewing Task)•Group 1>Group 2 (P<0.05),••Group 1>Group 3 (P<0.001) JRA with TMD**   - Jaw pain: 48.39•,•• - Ability to chew: 52.76•,•• - Quality of life: 46.04•,••   **JRA without TMD**   - Jaw pain: 33.5 - Ability to chew: 31 - Quality of life: 36.53   **Control group:**   - Jaw pain: 34.6 - Ability to chew: 33.04 - Quality of life: 34.5 | **Self-Report of Pain and Dysfunction: Correlation between Visual and Analog Scales(after chewing task)•P=0.001, ••P<0.01**  **JRA with TMD**   - Jaw pain: 0.97• - Ability to chew: 0.93• - Quality of life: 0.96•   **JRA without TMD**   - Jaw pain: 0.78• - Ability to chew: 0.59•• - Quality of life: 0.82•   **Control group:**   - Jaw pain: 0.97• - Ability to chew: 0.97• - Quality of life: 1.00•   **Changes in Analog Scale after Chewing Performance Tasks**  **•P<0.05**  **JRA with TMD**   - Jaw pain: 0.71 (0.48) - Ability to chew: 0.92 (0.36) - Quality of life: 1.00 (0.32)   **JRA without TMD**   - Jaw pain: 1.34 (0.18) - Ability to chew: 1.00 (0.32) - Quality of life: 1.00 (0.32)   **Control group:**   - Jaw pain: 2.39 (< 0.05)• - Ability to chew: 2.38 (< 0.05)• - Quality of life: 1.00 (0.32)   **Differences among Groups for Analog Self-Report Measures( After Chewing Task)•Group 1>Group 2 (P<0.05),••Group1>Group  2 (P<0.001),**  **†Group 1>Group 3 (P<0.05)**  **JRA with TMD**   - Jaw pain: 45.76• - Ability to chew: 49.78••,† - Quality of life: 37.81†   **JRA without TMD**   - Jaw pain: 32.42 - Ability to chew: 29.11 - Quality of life: 30.25   **Control group:**   - Jaw pain: 36.99 - Ability to chew: 36.12 - Quality of life: 27.66 | Greater incidence of TMD For the JRA group by gender was in females than males at a ratio of 2:1. |
| **1990**  **Honkanen et al.** | case-control | Finland | Total:137  Control:12  JCA:125  F:97  M:28  Age range:2-17  Mean age:8.8 | Not mentioned | Compare serum Chol, Vit A and E level and with disease activity. | N/A | **Lab test:**   - Vit A - Vit  E - Copper, - Zinc - Selenium - ESR - Total Chol | **Chol in F with JCA**   - (mean ±SD):4.69±0.97   **Chol in healthy F**   - ( mean ±SD):4.89±0.85   **Chol in M with JCA**   - (mean ±SD):4.62±0.88   **Chol in healthy M**   - ( mean ±SD):4.76±0.82 - Serum Vitamin E in JCA:22.8±5.2      - Serum Vitamin E in control:30.5±4.3 | **Chol in F**:   - JCA: 4.69±0.97 - Control: 4.89±0.85 - p-value: p<0.05   **Chol in M:**   - JCA: 4.62±0.88 - Control: 4.76±0.82 - p-value: Small sample size   **Vitamin E:**   - JCA: 22.8±5.2 - Control: 30.5±4.3 - p-value: p<0.001 - Significantly lower of total Cho in girls with JCA than healthy control. - chol correlated with the markers of disease activity by ESR and Hb(r=-.193,p=0.002 and r=0.208,p=0.02) - Possessive correlation between serum zinc and chol(r=0.227,p=0.002) - Significant correlation between Zinc and Vit A in serum( p<0.001) | N/A |
| **2002**  **C. M. Lofthose et al.** | case-control | UK | Total:44  JIA:22  Control:22  JIA:  F:17  M:5  Range 3-14 y | Paui-A:7   PA:15 | Determine the nutritional  status in JIA using anthropometric  Parameters and bioelectrical impedance analysis. | Determine the nutritional  Status in JIA on steroid with those who are not on steroid. | **anthropometric measurement**  **:**   - Weight - Height - HC - MAC - AMC, - Body fat - Total body water | **Height (cm):**   - JIA: (129.6,18.5) - Control :( 140.2, 22.) - p=0.096   **Weight (kg):**   - JIA:(28 ,9.4) - Control: (34.614.2) - p= 0.075   **HC:**   - JIA:(52.4,1.6) - Control: (53.1,2.1) - p=0.205   **MAC (cm)**   - JIA:(18.1,2.4) - Control: (21,3.2) - p=0.002   **AMC** (cm)   - JIA:(21.2,4.3) - Control: (26.1,5) - p= 0.001   **Body fat (%)**   - JIA:(15,2.3) - Control: (17.1,2.8) - p=0.009   **Total body water (kg)**   - JIA:(14.3,5.5) - Control: (18.6,7.3) - p=0.033 | - There is a significant difference between the JIA and controls for MAC (P<0.01(P=0.002) and AMC (P < 0.01 (P=0.009). - Significantly lower body fat percentage seen in Pauci-A than in controls (P=0.027). | Significantly lower values for MAC (P=0.018), AMC (P=0.046) and body fat percentage (P=0.028) in JIA on steroids compared with patients not receiving steroids.  significantly lower measurements seen in Patients not on steroids than the controls for MAC (P=0.044), body fat percentage (P=0.015) and total body water (P=0.048) |
| **2012**  **Christina F. Pelajo et al.** | cross-sectional | USA | Total: 154  F: 94  M:60  Age:2-19  Mean age: 10.6 ± 4.5 | OA:71  PA(R-):33  PA(R+): 4  Systemic-onset: 4  ERA: 29  Psoriatic: 13 | Examine the association between serum levels of 25-hydroxy vitamin D [25(OH) D] and disease activity in JIA. | Determine the prevalence of vitamin D deficiency and insufficiency according to  cut-offs of serum 25(OH)D (=19 ng/ml and 20-29 ng/ml) in JIA,   Determine factors associated with lower serum levels of 25(OH) D in JIA | **Assessment of child’s well-being**   - VAS - JADAS-27   **Lab test:**   - ESR - 25(OH)D - 25(OH)D3   **Anthropometric Measurements:**     - BMI | **Serum 25(OH)D levels(mean ± SD, range)**   - 25(OH)D:29.2 ± 9.2, 6 – 58 - 25(OH)D3: 27.9 ± 9.3,6 –58 - 25(OH)D2:1.3 ± 3.1, 0 – 29 - 25(OH)D ≤19: % (N): 13%(20) - 25(OH)D 20-29 : % (N):42% (64)   **JADAS-27(median, Interquartile range, Range):**   - Physician VAS: (1.2,0.2 –2.5,0 – 8) - ESR normalized: (0,0-0, 0-8) - Joint count: (2, 0-4, 0-18) - JADAS-27: (5.2,2-10.1,0-30.7) | - No association between   25(OH)D levels and  JADAS-27 (beta coefficient=0.002; 95%CI= -0.1, 0.1; p=0.97), nor with any of the four  Separate components of JADAS-27 (p-value range 0.32-0.89).     - Significant associations between JADAS-27 and JIA subtype (p=0.003), and ethnicity (p=0.006). | **Variables  influence 25(OH)D:**  **Season % (N)**   - Fall 32% (49) - Winter 25% (39) - Spring 23.5% (36) - Summer 19.5% (30)   **BMI category % (N)**   - Adequate 79%(122) - Obese 18% (28) - Underweight 3%(4) - BMI absolute value (median, IQR): 18.6, 16.2 – 21.9 - BMI percentile (median, IQR):   67, 41 – 88  **Vitamin D ingestion:**   - Dose of vitamin D3 on supplements in IU (median, IQR):400, 400 - 1,000 - Vitamin D ingested from the diet in IU (median, IQR): 239, 136 – 368 - Total daily vitamin D intake in IU (median, IQR): 368, 172 – 687 |
