# Supplementary material for: An investigation into the relationship between nutritional status, dietary intake, symptoms and health-related quality of life in children and young people with juvenile idiopathic arthritis: a systematic review and meta-analysis
Source: BMC Pediatr. 2023 Jan 2;23:3. doi: 10.1186/s12887-022-03810-4 (PMC9806873; doi:10.1186/s12887-022-03810-4)
Supplement: Supplementary file 3 — Additional file 3: Table 1. Characteristics of the selected studies (trials, exploratory, case and pilot studies). [file 12887_2022_3810_MOESM3_ESM.docx]

**Abbreviations for tables 1&2**

| AC | Arm circumference |
| --- | --- |
| Alb | Albumin |
| ALP | Alkaline phosphatase |
| AMC | Arm muscle circumference |
| AMA | Arm muscle area |
| ANA | Anti-nuclear anti body |
| BAP | bone alkaline phosphatase |
| BAP | Bone-specific alkaline phosphatase |
| BH | Body height |
| BI | Behavioural intervention |
| BSF | Biceps skin fold thickness |
| BUA | Broadband ultrasound attenuation |
| BW | Body weight |
| Ca | Calcium |
| CAHQ | Childhood health assessment questionnaire |
| Chol | Cholesterol |
| COC | Carboxylate Osteocalcin |
| Cr | Creatinine |
| CRP | C-reactive protein |
| D (25OHD) | 25-hydroxyvitamin |
| DAS 28 | Disease activity score 28 |
| DPA | Dual photon absorptiometry |
| DXA | Dual-energy x-ray absorptiometry |
| ERA | Enthesitis relate arthritis |
| ESC | Enhanced standard of care |
| ESR | Erythrocyte sedimentation rate |
| FMI | Total fat mass divided by height |
| GC | Glucortico therapy |
| Hb | Haemoglobin concentration |
| HC | Head circumference |
| HDL-C | High-density lipoprotein cholesterol |
| Ig | Immunoglobulin |
| IGF-1 | Insulin-like growth factor 1 |
| iPTH | Intact PTH |
| IQR | Interquartile range |
| JADAS-27 | Juvenile Arthritis Disease Activity Score 27 |
| JCA | Juvenile chronic arthritis |
| LDL-C | low-density lipoprotein cholesterol |
| LMI | Total lean mass divided by height |
| LS BMD | lumbar spine bone mineral density |
| MAC | Mid-arm circumference |
| MCP | Monocyte chemotactic protein |
| MMP | Matrix metalloproteinase |
| MTSM | Mean triceps skinfold measurement |
| MTX | Methotrexate |
| MUAF | Mid upper arm fat area |
| MUAM | Mid upper arm muscle area |
| NTX | N-telopeptide cross-links of collagen breakdown |
| OA | Oligoarticular |
| P | Phosphorous |
| PA | Polyarticular |
| Pacui-A | Pacuiearthicular arthritis |
| PEM | Protein-energy malnutrition |
| PF | Physical functioning |
| Pi | phosphate |
| PMC | Pre-arm muscle circumference |
| Pry and Pry-D | Pyridinolines |
| PSS | Psychosocial summary scores |
| PTH | Parathyroid hormone |
| PUFAs | Polyunsaturated fatty acids |
| RBP | Retinol binding protein |
| RDI | Recommended dietary intake |
| SDS | Standard deviation score |
| SFT | Skin fold thickness |
| SOS | Speed of sound |
| SSF | Sub scapular skinfold |
| TAC | Thickness Arm circumference |
| TBF | Total body fat |
| TB BMC | Total body bone mineral content |
| TC | Total cholesterol |
| TFM | Truncal fat mass |
| TG | Triacylglycerol |
| TIBC | Total iron-binding capacity |
| TMD | Temporomandibular Dysfunction |
| TMJ | Temporomandibular joint |
| TSF | Triceps skinfold |
| VAS | Visual analogue score |
| UAC | Upper-arm circumference |
| UAMA | Upper arm muscle area |
| ucOC | Undercarboxylated |
| WBC | White blood cell |
| WC | Waist circumference |
