# Supplementary material for: An investigation into the relationship between nutritional status, dietary intake, symptoms and health-related quality of life in children and young people with juvenile idiopathic arthritis: a systematic review and meta-analysis
Source: BMC Pediatr. 2023 Jan 2;23:3. doi: 10.1186/s12887-022-03810-4 (PMC9806873; doi:10.1186/s12887-022-03810-4)
Supplement: Supplementary file 4 — Additional file 4: Table 2. Characteristics of the selected studies (case-control, cross-sectional, cross-sectional with control and cross-sectional cohort). [file 12887_2022_3810_MOESM4_ESM.docx]

**Table 1:** characteristics of the selected studies (trials, exploratory, case and pilot studies)

| **Author**  **year** | **Study method** | **location of the study** | **age/gender** | **subtypes of arthritis** | **primary aim** | **secondary aim** | **measurements** | **Baseline values** | **intervention/exposure** | **primary outcome** | **secondary outcomes** |
| --- | --- | --- | --- | --- | --- | --- | --- | --- | --- | --- | --- |
| **2016**  **Berntson, Lillemor et al** | Exploratory study | Sweden | Total JIA**:**7  F:3  M:4  Age: 3.2-11.1 | Poly RF-:3  ERA:3  PA: 1 | To explore the immediate anti-inflammatory  effect of EEN in children with JIA. | N/A | Lab test:   - ESR - Thrombocytes - faecal calprotectin - 92 proteins involved   In inflammatory processes   - JADAS‑27 - number of - active joint - global assessment VAS - morning   stiffness   - CHAQ | JADAS‑27: p=0.016  number of active joints: p= 0.031  morning  stiffness: p= 0.031  MMP-1  decreased significantly (p=0.047), as did MCP-4 (p=0.031) and 4E-BP1  (p=0.031). | EEN was given in duration of 3-8 weeks. | EEN have an  immediate anti-inflammatory effect**,** reduced inflammatory  proteins, resulting in clinical improvement  in patients with JIA | N/A |
| **2019**  **TANG et al** | Randomised control trial | China | Total:36  JIA:18  Control:18  JIA: 7.6  Control: 6.3  JIA: F: 56%  M: 44%  Control:  F: 62%  M: 28% | JIA | Examine the effect of cholecalciferol supplementation on serum vitamin D levels, disease activity scores, and bone mineral density in patients with JIA. | N/A | Lab test:   - ESR - serumVit D - Parathyroid hormone levels   JADAS‑27  Bone mineral density (BMD) | **25OHD**  Experimental group:33.29 (12.87)  Control group: 51.60 (34.77)  P=0.15  **JADAS-27**  Experimental group:16.19 (9.57)  Control group: 14.31 (6.22)  P=0.84  **Z‑score**  Experimental group :‑1.10 (1.48)  Control group: ‑1.15 (0.96)  P= 0.94 | Cholecalciferol Vitamin D supplementation  2000 IU per day.for 24 weeks | **25OHD after** 24 weeks:  Experimental group: 69.25  Control group: 38.83  P<0.05  **JADAS-27**  Experimental group: 0.94  Control group:1.06  P>0.05  **Z‑score**  Experimental group: ‑0.61  Control group: -0.97  P>0.05  No correlation between vitamin D, disease activity and BMD. | N/A |
| **2016**  **Berntson, Lillemor et al** | pilot study | Sweden | F:7.4 years old  Diagnosed at age of:3.2 | PA | To find out how the EEN treatment influenced the microbiome and metabolome in JIA patient. | To assess active joints, joint with limited range of motion and child health assessment. | Lab test:  •Faecal analysis  •RF  •ANA  •anti-CCP,  •HLA-B27  questionnaire:  •CHAQ  Clinical exam:  •Number of inflamed joints  •Morning stiffness  •Pain (VAS 0-100 mm)  •assessment of disease activity(VAS 0-100) | Active joints: 6  Joints with limited range of movement: 4  CHAQ: 7  •Faecal microbiota results: high Bacteroidetes | EEN was given in two periods of 6.5 weeks each, several months apart. | Faecal microbiota results: lower proportion of  Bacteroidetes during treatment with EEN. | After administrating EEN:  Active joints: 2  Joints with limited range of movement: 3  CHAQ: 0 |
| **2014**  **Berntson, Lillemor et al** | case study | Sweden | F:7.4 years old  Diagnosed at age of:3.2 | PA | Explore if EEN had an anti-inflammatory effect in JIA. | N/A | Lab test:  • ESR  •CRP  •ANA  • anti-CCP  • RF  •HLA-B27  Questionnaire  •CHAQ   - JADAS27   Physical exam:   - VAS disease activity assessment | VAS score:  •Global assessment doctor report:61    •Global assessment parents report:80  •Pain according to a parent: 55  Morning stiffness: 48 | EEN was given in two periods of 7weeks each, several months apart, during the year of the study. | Treatment with EEN had a remarkable effect that persisted for months. | N/A |
| **2006**  **LORI J. STARK et al.** | Randomized clinical trial | USA | Total:49  BI group:25  F:73%  ESC group:24  F: 79%  Age:4-10  BI group: 6.1 ± 2.0  ESC group: 6.8± 2.0 | Pauci-A  BI :50%, ESC: 62.5%  PA: BI :31%,  ESC: 25%  Systemic:  BI :11% , ESC: 12.5%  Mono arthritis: BI :8%, ESC: 0  Inflamed joints(no.):  SD=Mean  BI:1.6 + 2.3  ESC:2.0 + 2.8 | Examine a behavioural intervention (BI) to increase calcium intake in JRA. | Investigate the impact of the BI on the bone mass and maintenance of Ca intake within 6 and 12 months follow up after treatment. | Dietary evaluation:  •7 consecutive days of diary.  Bone mass measured:  • x-ray of L1-L4, TB BMC,AL BMD,LSBMD  Anthropometric measurements:  •Weight  •height  Dia Sorin 25-OH-D assay:  •Vit D | Total calcium (mg): BI  •Baseline: 972 ±372  •6 months: 1586 ± 406  •12 months: 1547 ± 463  Total calcium (mg): ESC  •Baseline: 961±438  •6 months: 1395± 565  •12 months: 1351± 444  •P-value (for BI and ESC): 0.9  Average serum 25-hydroxyvitamin D (25-OH-D(BI):  •Baseline: 32 ± 10 (16–57)  •6 months: 35 ± 9 (19–51)  •12 months: 37 ± 8 (25–52)  Average serum 25-hydroxyvitamin D (25-OH-D(ESC):  •Baseline: 36± 12 (20–60)  •6 months: 35 ± 13 (20–72)  •12 months: 32 ± 8 (22–52)  •P-value (for BI and ESC): >.05 | Group 1: JRA received the 6-session BI increased to increase taking dietary calcium  Group 2: JRA received 3-session ESC to increase Ca. | • Ca was significantly greater in the BI group than their baseline and at both the 6- (P <.0001) and 12-month follow-up (P < .001).  •25-OH-D concentration did not differ between or within intervention group p >.05  • BI group had a greater increase in TB BMC over time compared with the ESC group (P =.002). | Total energy (kcal):  BI:  Baseline: 1560± 374  6months: 1678 ± 460  12 months: 1784 ± 431  ESC:  Baseline:1628± 420  6months:1775 ± 432  12 months:1858 ± 396  P=.09  Protein (g):  BI:  Baseline:52± 19  6months:61 ± 18  12 months:65 ± 19  ESC:  Baseline:58 ± 18  6months:62 ±18  12 months:65 ± 18  P=0.58  Carbohydrates (g):  BI:  Baseline: 218 ±56  6months: 246± 108  12 months: 246±63  ESC:  Baseline:222 ± 58  6months:243 ±62  12 months:258± 58  P=0.75  Fat (g):  BI:  Baseline: 55± 18  6months: 59± 21  12 months: 61± 19  ESC:  Baseline: 58 ± 21  6months: 66 ± 24  12 months: 65 ± 19  P=0.95 |
| **2015**  **Giancarla Dilandro et. Al** | Control trial | Italy | Total:22  JIA:12  Age:3-10 mean age:5.5  Control:10  Ages:4-10  Mean age:7  gender not mentioned | JIA | Assess the effects of a scheduled dosage of biscuits with iron in JIA. | N/A | Blood test  •ESR  •CRP  •Hb  •Albumin  •Transferrin  •Ferritin  •Serum iron  3-days food record  Anthropometric  Parameters:  •Height  •Weight  •BMI | Baselines:  Group1:  •weight:18.9 (12.5–33.0) kg  •BMI: 15.1 (12.5–17.8)kg/m2  Group2:  •weight: 23.6 (14.9–44.0) kg  •BMI: 15.8(13.8–21.4) kg/m2  Before intervention:  group 1(G1),group2(G2)  Median,p-value  Hb (g/dl):  •G1:12,  •G2: 13.3,  •P=0.016  Serum iron (jig/dl)  •G1:48.5  •G2:68  •P=0.003  Transferrin (mg/dl)  •G1:275.5  •G2:453  •P=0.947  ESR (mm/h)  •G1:20.5  •G2:58  •P=0.692  Ferritin (ng/ml)  •G1:24.6  •G2:223  •P=0.21  CRP (mg/dl)  •G1:0.64  •G2:22.2  •P=0.106  Albumin (g/dl)  •G1:3.8  •G2:4.4  • P= 0.478 | Biscuit supplementation (4 months)  •Energy (kcal)  5g:21  60g: 252  •Total fat  5g:0.4  60g: 4.8  •Saturated fat  5g:0.2  60g: 2.4  •Monounsaturated fat  5g:0.15  60g:1.8  •Polyunsaturated fat  5g:0.06  60g: 0.72  •Total carbohydrates  5g:3.8  60g: 45.6  •Sugars 5g:1.3  60g: 15.6  •Fiber:  5g:0.2  60g: 2.4  •Protein  5g:0.4  60g: 4.8  •Sodium  5g:0.033  60g:0.369  •Thiamine  5g:0.03  60g: 0.36  •Riboflavin  5g:0.04  60g:0.48  •Niacin  5g:0.45  60g: 5.4  •B6-vitamin  5g:0.04  60g: 0.48  •Calcium  5g:15.5  60g: 186  •Iron fumarate  5g:0.3  60g: 3.6 | After intervention:  Group1:  •Weight:19.5 (13.0–34.0) kg 15.7  •BMI:(12.0-18-0) kg/m2  Group2:  •Weight: 24.7 (15.0–48.0) kg  •BMI: 16.2 (13.8–21.9) kg/m2  Hb (g/dl)  •G1:12.4  •G2:13  •P= 0.005  Serum iron (jig/dl)  •G1:52.5  •G2:68.5  •P= 0.166  Transferrin (mg/dl)  •G1:266.5  •G2:282.5  •P= 0.895  ESR (mm/h)  •G1: 10  •G2:17.5  •P= 0.002  Ferritin (ng/ml)  •G1:25.6  •G2:21.2  •P= 0.621  CRP (mg/dl)  •G1:0.13  •G2:0.14  •P= 0.112  Albumin (g/dl)  •G1:4.1  •G2:5  •P= 0.438 | N/A |
| **2014**  **Jeff Golini and Wendy Lou Jones** | Clinical trial | Bulgaria | Total:16  Age:<17  Mean age: 13.3  gender not mentioned | Pauci- A  PA  systemic onset | Evaluate the safety and efficacy of a nutritional supplement (Kre-Celazine) in the setting of inflammatory JRA/JIA. | Evaluate the effect of nutritional supplements (Kre-Celazine) in pain and range of motion. | Blood test:  •ANA  •ESR  •CRP  Pain assessment:  • pain scale ( 0-10) | Baseline pain scores: by pt:3  •ANA – 1:640  •CRP – 9 mg/L  •ESR – 22mm/hr  Baseline pain scores: by pt: 3&4  •ANA – 1:640  •CRP – 8 mg/L  •ESR – 18mm/hr  Baseline pain scores by pt: 3&4  •ANA – 1:320  •CRP – 5 mg/L  • ESR – 19mm/hr  Baseline pain scores by pt: 3&5  •ANA – 1:1,280  •CRP – 18 mg/L  •ESR – 32 mm/hr  Baseline pain scores by pt:4  •ANA – 1:1,280  •CRP – 11 mg/L  •ESR – 28mm/hr  Baseline pain scores by pt:2  •ANA – not reported  •CRP – 7.04 mg/L  •ESR – 3 mm/hr  Baseline pain scores by pt:2  •ANA – not reported  •CRP – normal  •ESR – 6 mm/hr  Baseline pain scores by pt:2  •ANA – normal  •CRP – normal  •ESR – normal  Baseline pain scores by pt:2  •ANA – normal  •CRP – normal  •ESR – normal  Baseline pain scores by pt: 2&1  •ANA – normal  •CRP – 26.4mg/L  •ESR – 39 mm/hr  Baseline pain scores by pt:3  •ANA – 1:160  •CRP – normal  •ESR – normal  Baseline pain scores by pt: 4&5  •ANA – normal  •CRP – 103 mg/L  •ESR – 50 mm/hr  Baseline pain scores by pt:3  •ANA – normal  •CRP – 30 mg/L  •ESR – 12.2 mm/hr  Baseline pain scores by pt:3  •ANA – 1:160  •CRP – normal  •ESR – normal  Baseline pain scores by pt:3  •ANA – 1:160  •CRP – normal  •ESR – normal  Baseline pain scores by pt: 2&3  •ANA – normal  •CRP – 99.77 mg/L  •ESR – 40mm/hr | Each participant:  Two 750 mg capsules (total1,500 mg) of Kre Celazine, an oral for non-prescription, nutritional  Supplement composed of a proprietary alkali buffered, creatine monohydrate and acetylated fatty acids mixture, for a period of 30 consecutive days. | Final pain scores by pt: 0&1  •CRP – 5 mg/L  • ESR – 5 mm  Final pain scores by pt: 0&0  •CRP – 4 mg/L  •ESR – 6mm  Final pain scores by pt: 0&0  •CRP – 4 mg/L  •ESR – 10mm  Final pain scores by pt: 0&1  •CRP – 6 mg/L  •ESR – 20mm  Final pain scores by pt:1  •CRP – 6 mg/L  • ESR – 16mm  Final pain scores by pt:1  •CRP – 2.18 mg/L  • ESR – 3 mm/hr  Final pain scores by pt :0  •CRP – normal  •ESR – 7 mm/hr  Final pain scores by pt: 0  •CRP – normal  •ESR – normal  Final pain scores by pt:1  •CRP – normal  •ESR – normal  Final pain scores by pt: 1&0  •CRP – 20 mg/L  •ESR – 19 mm/hr  Final pain scores by pt: 0  •CRP – normal  •ESR – normal  Final pain scores by pt:0&1  •CRP – normal  •ESR – 25 mm/hr  Final pain scores by pt:2  •CRP – normal  •ESR – 12 mm/hr  Final pain scores by pt: 0  •CRP – normal  •ESR – normal  Final pain scores by pt:2  •CRP – normal  •ESR – normal  Final pain scores by pt:2&1  •CRP – 16 mg/L  •ESR – 20 mm/hr | Range of motion:  Reported as normal. |
| **2012**  **Tamer Gheita et**  **Al.** | Control-trial | Egypt | Total:47  JIA:27  Control:20  Mean age: JIA  12.78±3.27  F:19  M:8  Mean age: control  12.15±3.12 | Systemic onset:8  Pauci-A:9 (persistent:3 extended:5)  PA(RF-):10 PA(R+):3 | Demonstrate the effect  of ω-3 FAs supplements on the serum IL-1 and TNF-a  levels, the clinical manifestations, laboratory investigations,  disease activity, functional capacity, and response criteria. | The change in daily requirement of NSAID in JIA. | Clinical exam  lab test:  •Serum IL-1  •TNF-α level  Disease activity:  •JADAS-27Questionnaire:  CHAQ | TNF-α:  •Base line: (90.85±6.54 pg/ml)  •After intervention:  7.46±3.14 pg/ml  •p<0.001  IL-1 level  •Baseline: 140.89±13.47  •After intervention: 18.07±4.4 pg/ml  •p<0.001  Mean active joint count  •Baseline: 4.15±3.08  •After intervention: 0.59±0.84  •p<0.001  JADAS-27  •Baseline: 18.63±6.82  •After intervention: 8.4±3.18  •p<0.001  CHAQ  •Baseline: 0.7±0.47  •After intervention: 0.11±0.32  •p<0.001  ESR  •Baseline: 50.44±25  •After intervention: 26.65±12.87  •p<0.001 | Dietary supplements of omega-3 FAs (2 g) in a dose of 2 soft gelatine capsules per day for 12 weeks. Each capsule contains 1,000 mg omega -3 with 50% essential fatty acids, 300 mg EPA, and 200 mg DHA. | •Steroid dose significantly negatively correlated with both the TNF-α and IL-1 (r=−0.41, p=0.03).    •TNF-α negatively significantly correlated with the ESR and JADAS-27 (r=−0.56, p=0.002, and r=−0.4, p=0.038, respectively) which became insignificant at the end of the study (r=−0.07, p=0.75, and r=−0.28, p=0.16, respectively). | NSAIDs was  reduced in all patients after 12 weeks on omega-3 FAs  Supplementation (88.88% of them stopped taking NSAIDs). |
| **2018**  **Nataliia Yarema et al.** | Randomised controlled trial | Ukraine | Total:68  JIA:53  Control:15  Age: 5-16 years.  Gender not mentioned. | Not mentioned | Evaluate the influence of ω-3 PUFA on inflammatory processes in children with RA. | N/A | **Lab test:**   - IL-1 - IL-4 - IL-6 - CD3 - CD4 - CD8 - CD16 - CD22 - CIC - Ig M - IgG - Ig A   **clinical examination:**   - Disease activity  by DAS 28 - Tenderness and swelling                 by VAS (    0–10) | **RA(backbone therapy):**   - IgA before treatment: 3.18 ± 0.12 - IgM before treatment: 2.57 ± 0.16 - IgG before treatment: 14.63 ± 0.43 - IgA after treatment: 2.27 ± 0.14 - IgM after treatment: 1.94 ± 0.11 - IgG after treatment: 12.10 ± 2.11   **RA(modified therapy):**   - IgA before treatment: 2.96 ± 0.08 - IgM before treatment: 2.55 ± 0.14 - IgG before treatment: 13.38 ± 0.23 - IgA after treatment: 1.96 ± 0.02 - IgM after treatment: 1.64 ± 0.05 - IgG after treatment: 10.97 ± 0.11   **Control group:**   - IgA: 1.90 ± 0.12 - IgM: 1.43 ± 0.22 - IgG: 10.02 ± 0.54   **IgA, g/l:**   - P1: ≤ 0.001 - P2: ≤ 0.001 - P3: ≤ 0.01   **IgM, g/l:**   - P1: ≤ 0.001 - P2: ≤ 0.001 - P3: ≤ 0.01   **IgG, g/l:**   - P1: > 0.05 - P2: ≤ 0.001 - P3: > 0.05 | **Group 1:**  20 backbone therapy: 1  the capsule contained 1,000 mg of ω-3 acids,300 mg of  eicosapentaenoic acid (C20:5 ω-3) and 200 mg of docosahexaenoic  acid (C22:6 ω-3), 498 mg of other fatty acids and 2 mg of  d-α-tocopherol  **Group 2** :  13 modified therapy:  backbone therapy and Epadol(Kyiv Vitamin Factory) | - Both groups had an improved immune status. - significantly           improved parameters: (≤ 0.05)   - IgA - IgM - CD8 - CD4/CD8 - CD22 - IL-1 - IL-4 - CIC | N/A |
